# Comparison of FDG and FMISO uptakes and distributions in head and neck squamous cell cancer tumors

**DOI:** 10.1186/s13550-021-00767-w

**Published:** 2021-04-14

**Authors:** Sadek A. Nehmeh, Mohamed B. Moussa, Nancy Lee, Pat Zanzonico, Mithat Gönen, John L. Humm, Heiko Schöder

**Affiliations:** 1 Medical Physics, Memorial Sloan-Kettering Cancer Center, New York, NY USA; 2 Chemistry Department, Stony Brook University, Stony Brook, NY USA; 3 Radiation Oncology, Memorial Sloan-Kettering Cancer Center, New York, NY USA; 4 Memorial Sloan-Kettering Cancer Center, New York, NY USA; 5 Memorial Sloan-Kettering Cancer Center, New York, NY USA; 6 Weill Cornell Medical College, New York, NY 10021 USA

**Keywords:** FDG, FMISO, Glucose metabolism, Hypoxia

## Abstract

**Purpose:**

Glycolysis is increased by hypoxia, suggesting a possible correlation between the accumulation of *2-[18F]fluoro-2-deoxy-D-glucose *(FDG) in malignant tumors and regional hypoxia defined by 1H-1-(3-[18F]fluoro-2-hydroxypropyl)-2-nitroimidazole (FMISO) PET. The aim of this study is to investigate the intra-tumoral spatial distribution and quantitative relationship between FDG and FMISO in a cohort of head and neck squamous cell cancer (HNSCC) patients.

**Methods:**

Twenty HNSCC patients with 20 primary tumors and 19 metastatic lymph nodes (LNs) underwent FDG and FMISO PET within 1 week. The metabolic target volume (MTV) was defined on the FDG PET images using a region growing algorithm. The hypoxic volume (HV) was defined by the volume of voxels in an FMISO image within the MTV that satisfy a tumor-to-blood ratio (T/B) greater than 1.2. FDG and FMISO lesions were co-registered, and a voxel-by-voxel correlation between the two datasets was performed. FDG and FMISO TVs’ SUVs were also compared as well as the intra-tumoral homogeneity of the two radiotracers. Separate analysis was performed for the primary tumors and LNs.

**Results:**

Twenty-six percent of the primary tumors and 15% of LNs showed a strong correlation (R > 0.7) between FDG and FMISO intra-tumor distributions when considering the MTV. For the HV, only 19% of primary tumors and 12% of LN were strongly correlated. A weak and moderate correlation existed between the two markers SUV_avg_, and SUV_max_ in the case of the primary tumors, respectively. However, this was not the case for the LNs. Good concordances were also observed between the primary tumor’s and LNs HV SUV_avg_s as well as between the corresponding hypoxic fractions (HF’s).

**Conclusions:**

A moderate correlation between FDG and hypoxia radiotracer distribution, as measured by FMISO, seems to exist for primary tumors. However, discordant results were found in the case of LNs. Hypoxia appears to be the dominant driver of high FDG uptake in selected tumors only, and therefore FDG PET images cannot be used as a universal surrogate to identify or predict intra-tumor hypoxia.

## Introduction

Tumor hypoxia is an independent prognostic indicator of treatment outcome for several malignancies. Hypoxic tumors, in general, express a more aggressive phenotype, are radioresistant, and therefore, have an increased likelihood of locoregional recurrence, develop distant metastasis, and have an overall poor outcome [1]. Fluoromisonidazole (FMISO) PET has been recognized as a noninvasive method for detecting tumor hypoxia in several types of solid tumors, including HNC [2]. FMISO retention was shown to correlate with tissue hypoxia as assessed by pO2-polarography [3–5], Likewise, it was also correlated with molecular biomarkers of hypoxia [6]. In a prospective trial of HNSCC patients undergoing chemoradiation, HIF1α expression correlated with an increase in intratumoral FMISO uptake during the first 2 weeks of chemoradiation, and high levels of HIF1α and CAIX were associated with a delayed resolution of the FMISO uptake between weeks 2 and 5 [6].

Tumor FDG uptake is based on tumor hyperglycolysis (upregulation of glucose transporters (GLUTs) and glycolytic enzymes) [7, 8]. Both of these can be driven by hypoxia-inducible factor-1 (HIF-1) transcription, which is activated under tumor hypoxia [9]. Furthermore, both FMISO clearance from the vasculature and its rate of trapping in hypoxic tissues are slow. Consequently, the image contrast of malignant tumors using radio-labeled nitroimidazole derivatives is low compared to that achieved with FDG PET. This is exacerbated in tumors with low hypoxic fractions due to partial-volume effect. Moreover, the synthesis of FMISO is currently not widely available, usually requiring the presence of in-house radiopharmacy expertise, which is not available to most nuclear medicine departments. Therefore, it would be practical if hypoxia information could be derived from a routinely available radiotracer such as FDG.

Here, we investigate to what degree hypoxia, as imaged by FMISO, contributes to FDG uptake, surrogate of glucose metabolism, in malignant HNSCC tumors. Analysis is carried out using global metrics (SUVmax, SUVavg) as well as for the intra-tumoral distribution using voxel-wise correlation. Separate analysis is carried out for primaries and LNs. We also report on the general similarity of the two radiotracers’ distributions within the MTV by means of comparing the corresponding activity volume histograms (AVHs).

## Methods

### Patient data

This study was approved by the Institutional Review Board at Memorial Sloan Kettering Cancer Center (MSKCC), New York, NY, and all subjects signed a written informed consent. FMISO was produced under after RDRC approval by the cyclotron at MSKCC. A total of 20 HNSCC patients (19 males and 1 female) scheduled for definitive radiotherapy (RT) were enrolled in this study. Nineteen patients had squamous cell carcinoma of the oropharynx, and one patient had squamous cell carcinoma of the larynx. Subjects were accrued from two clinical trials that were approved by the local medical ethical committee. All subjects included in the study had histologically proven head and neck squamous cell carcinoma, and none of them prior radiation therapy or chemotherapy treatment for this diagnosis. The first 14 patients were enrolled from a static FMISO imaging protocol, while the other 6 were included in an amended protocol using dynamic FMISO imaging. In the latter case, FMISO data from only the last frame of the dynamic dataset (~ 160 min post-injection) were used in the current study. All subjects underwent a pretherapy FDG PET/CT scan, followed by FMISO PET/CT scan within one week of the FDG PET study and preceding therapy. For the latter scans, peripheral venous blood samples were taken and radio-assayed in a scintillation well counter calibrated for ^18^F and the blood activity concentrations derived in order to estimate the FMISO tumor/blood (T/B) ratio.

The average age of the 20 patients was 57 years (range 25–79 years). In one study (Patient 1), the blood sample coagulated; therefore, correlation between FDG and FMISO using voxels satisfying a T/B ratio greater than 1.2 was not performed. This threshold value of 1.2 was an operational definition used to segment hypoxic from non-hypoxic voxels. It arose from a statistical analysis of whole-body FMISO PET image data wherein it was observed that < 5% of voxels corresponding to normal tissues exceeded this value. Patient age, primary disease site, disease stage, and FDG and FMISO scan times post-injection are summarized in Table 1. All LNs that met the pathologic criteria were included in this study. The mean MTVs for the primary tumors and LNs were ∼10 cm^3^ (range 4–35 cm^3^) and ~ 16 cm^3^ (range 1–37 cm^3^), respectively. Table 1 also summarizes the HPV analysis results for the 7 patients out of the 20 patients included in this study. For the other 13 patients, HPV analysis was not performed.

**Table 1:** Patients demographics

Demographics							
Patient #	Sex	Age	Primary Location	Stage	^FDG^ Time PI	^FMISO^ Time PI	HPV
1	M	79	Oropharynx	T3N2c	91	171	–
2	M	67	Oropharynx	T3N1	82	193	–
3	M	48	Oropharynx	T1N1	65	181	–
4	M	54	Larynx	T2N2bM0	74	157	–
5	M	62	Oropharynx	T4N2c	131	147	–
6	M	60	Oropharynx	T2N1	83	153	–
7	M	64	Oropharynx	T2N2b	71	166	–
8	M	60	Oropharynx	T4N2c	50	180	–
9	M	62	Oropharynx	T4aN2c	69	196	–
10	M	55	Oropharynx	T2N1	85	115	–
11	M	66	Oropharynx	T1N3	180	155	–
12	M	56	Oropharynx	T3N2c	85	157	–
13	M	47	Oropharynx	T3N1	68	153	–
14	M	57	Oropharynx	T2N2c	75	160	HPV+
15	M	25	Oropharynx	T2N2bM0	85	156	HPV+
16	M	56	Oropharynx	T2N2bM0	63	154	HPV+
17	F	63	Oropharynx	T2N2bM1	80	182	HPV+
18	M	57	Oropharynx	T1N2b	66	148	HPV+
19	M	49	Oropharynx	T2N2b	68	135	HPV−
20	M	58	Oropharynx	T2N2b	77	181	HPV+

### PET/CT acquisition

Patients were scanned in a supine position on a flat-top couch insert. The head, neck, and shoulders were immobilized using an Aquaplast mask prepared during the RT simulation session. To minimize patient misalignment during the multiple studies, marks were drawn on the flat insert to ensure proper repositioning of the immobilization hardware. In addition, small CT markers were used on the patient's immobilization mask to assist in the image registration. All PET and CT images were acquired using either a General Electric (G.E. Medical Systems, Waukesha, WI) Discovery LS PET/CT scanner (first 14 patients) or a Discovery STE PET/CT scanner (last 6 patients). Studies that were performed on the DLS were acquired in 2D mode, while those performed on the DSTE were acquired in 3D mode. PET emission data were corrected for attenuation, scatter, and randoms, and then iteratively reconstructed using the standard clinical FDG reconstruction parameters (28 and 20 subsets for the DLS and DSTE, respectively, 2 iterations, post-filter, 6.0 mm full width at half maximum, loop filter, 4.3 mm full width at half maximum). In all cases, CT images were acquired with no IV contrast and using the following settings: 120 kVp, 80 mA, and 4.25 mm and 3.3 mm slice thicknesses for the DLS and DSTE, respectively.

### FDG PET/CT protocol

Patients were injected intravenously with an average of 532.8 MBq (range 458.8–610.5) of [^18^F]-FDG after a fasting period of 6 h. PET scans were acquired for 3 min/bed position at 80 ± 28 min (range 50–180) post-injection (P.I.).

### FMISO PET/CT protocol

Patients were injected intravenously with an average of 387.8 MBq (range 203.5–451.4) of [^18^F]-FMISO. A fasting state prior to the FMISO study was not required. For the first cohort of patients, PET data were acquired at a mean P.I. time of 163 min (range 114 min to 195 min), while for the second cohort, the last frame was acquired at an average of 159 min P.I. (range 135 min to 181 min). In both cases, FMISO data were acquired over one PET bed position with the lesion at the center of the field of view (FOV) and then scanned for 8 and 10 min/bed position, respectively. Venous peripheral blood samples were obtained immediately after the FMISO PET/CT session.

### Image analysis

The FMISO and FDG scans were spatially registered using the GE AW workstation. Image registration was first performed by aligning the corresponding CT datasets. Then, the target volumes in the FMISO CT and FDG CT (reference image set) image sets were rigidly registered by mutual information maximization, followed by a local registration of the target volumes. This was performed on a lesion-by-lesion basis. The transformation matrices were then applied to the FMISO image sets, thus registering the FMISO target volume to that of FDG. The imaging data, initially in units of microcuries per milliliter, were decay-corrected to the time of injection and converted into SUV. The FDG PET MTV (i.e. metabolic target volume) was then segmented by a physicist with PET lesion segmentation expertise using the GE region-growing algorithm provided on the AW workstation, and the corresponding TV was measured. A mask, corresponding to the FDG TV, was created, and the corresponding volume in the spatially registered FMISO PET image set was segmented. The coordinates and SUV of each voxel in the FDG and FMISO PET TV s were then extracted to text files using ImageJ [10]. For the convenience of presenting T/B threshold ratios for hypoxia tracer uptake, blood activity concentration was also converted to SUVs. Blood SUVs were determined using the measured blood aliquots per unit weight acquired at the time of the PET scan, decay-corrected to the time of injection and divided by the administered activity per unit body weight.

The relationship between the FDG and FMISO distributions within the TV was analyzed using a voxel-by-voxel SUV correlation between the registered tumor volumes. The corresponding Pearson correlation coefficient (R) was calculated using two criteria: First, for all the voxels included in the TV, and second, for voxels within the FMISO TV satisfying a T/B ≥ 1.2. The strength of the correlation between the FDG and FMISO distributions was defined according to the following criteria: R < 0.5 indicates a weak correlation, 0.5 ≤ R < 0.7 indicates a moderate correlation, and R ≥ 0.7 a strong correlation. The hypoxic fraction volume (HFV), using a T/B ratio of 1.2, was defined as

1 $$HFV=\frac{HV}{MTV}$$

The FDG and FMISO AVHs, defined as the percent of total tumor volume with SUV greater than a SUV threshold, were also calculated and compared on a lesion-by-lesion basis. For this comparison, the maximum SUVs for both the FDG and FMISO were normalized to 100%.

## Results

**Fig. 1:**
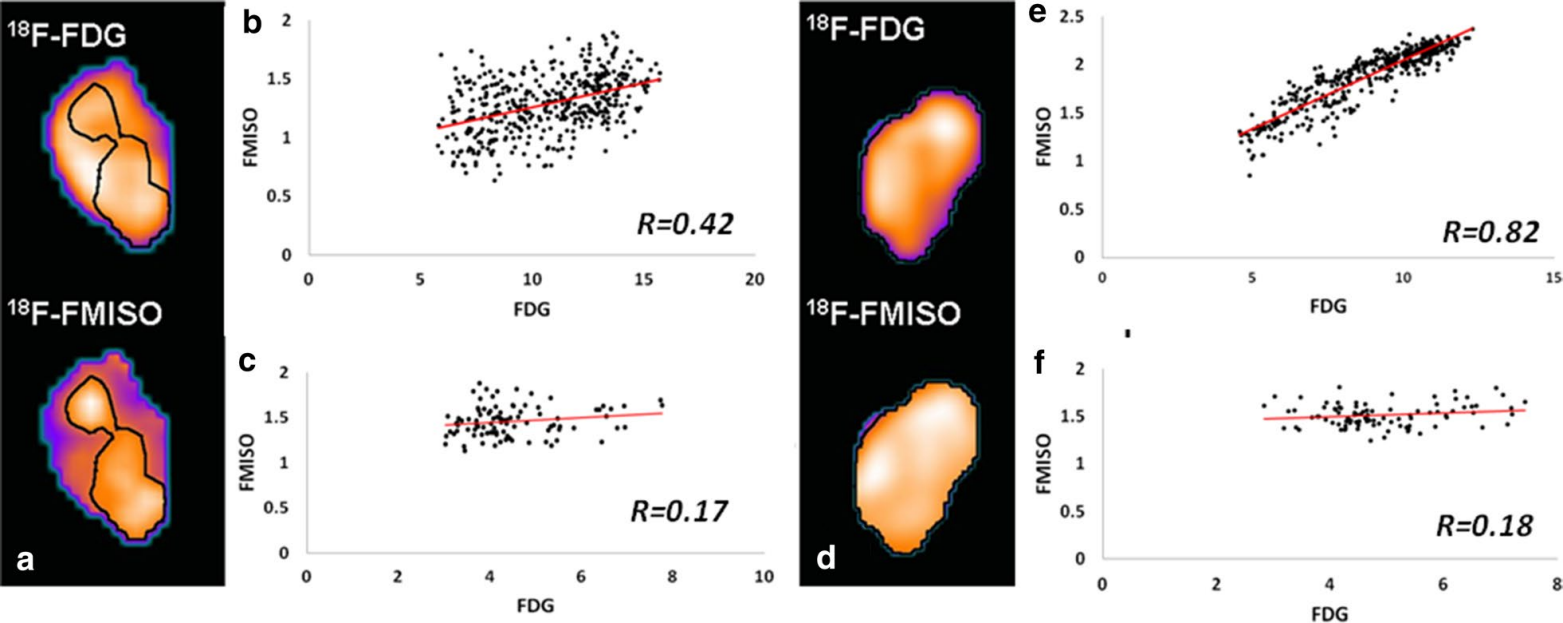
Example of FDG-SUV vs FMISO-SUV scattergrams for a **b** low correlation lesion, R = 0.37 (pt# 12) and **e** a strong correlation lesion, R = 0.76 (pt# 16). Figures **a**, **d** are the corresponding FMISO and FDG coronal images; the black contour refers to the HV satisfying a FMISO T/B > 1.2. For reference, FDG vs FMISO scattergrams for normal brain tissue regions are shown in figures **c**, **f** for the two patients, respectively

Figure 1a, d present examples of the central coronal PET slices corresponding to the FMISO and FDG TV images for poor and well-correlated datasets, respectively. The hypoxic TVs, defined by a T/B greater than 1.2, are overlaid (black contour). The corresponding voxel-by-voxel SUV FMISO-versus-FDG scattergrams are displayed in Fig. 1b, e, respectively. For reference, scattergrams corresponding to normal brain tissues for each patient are shown in Fig. 1c, f, respectively.

The Pearson correlation coefficients (R) between FDG and FMISO intra-tumor distributions, on a voxel-by-voxel basis, for all 20 patients, for both MTV and the HV, along with the FDG MTV (primaries: 9.99 ± 7.327 cc; LN: 15.60 ± 10.72 cc), maximal tumor SUV, blood SUV, and HFVs are summarized in Tables 2 and 3 for the primary tumors and the LNs, respectively. Tables 2 and 3 also report for each FDG MTV whether the SUV_max_ voxel falls within the corresponding FMISO HV (Y) or not (N). The average Pearson correlation coefficient between FDG and FMISO intensities using voxels included within the volume defined by the MTV was 0.55 (range 0.11–0.81) and 0.51 (range 0.31–0.88) for the primary tumors and LNs, respectively, with 26% of the primary tumors and 15% of the LNs, showing a strong correlation R > 0.7. However, for the HV, the average correlation coefficient between FDG and FMISO SUVs was only 0.34 (range 0.21 to 0.71) and 0.38 (range 0.01 to 0.87) for the primary tumors and LNs, respectively, with 19% of the primary tumors and 12% of the LNs showing a strong correlation between the two radiotracers intra-tumor distributions. The average HFV was 63% (range ∼0.28% to∼100%) and 59% (range 3% to 100%) for the primary tumors and LNs, respectively. When the MTVs were considered, the primary tumors showed a weak (R = 0.15) and weak (R = 0.36) correlations between the FDG and FMISO average (data not shown) and maximum (data not shown) tumor SUVs.

**Table 2:** Results summary for the primary tumors LNs

Primary Tumors														
Patient #	FDG MTV	Blood SUV	FDG SUV_max_	FDG SUV_avg_	FMISO SUV_max_	FMISO SUV_avg_	FDG SUV_avg_ (T/B > 1.2)	FMISO SUV_max_ (T/B > 1.2)	FMISO SUV_avg_ (T/B > 1.2)	HV	HF	R	R (T/B > 1.2)	FDG SUVmax within FMISO HV2
1	13.01	N/A	17.33	10.54	2.88	2.02	–	–	–	NBD5	–	0.66	–	N
2	16.82	1.52	12.72	7.64	3.69	2.09	8.13	3.69	2.41	11.30	67.21	0.49	0.31	Y
3	NFU6	–	–	–	–	–	–	–	–	–	–	–	–	Y
4	4.21	1.38	7.62	4.83	1.86	1.38	6.52	1.86	1.74	0.56	13.28	0.57	0.64	Y
5	12.89	2.10	32.24	19.08	3.69	2.52	22.33	3.69	2.98	6.23	48.34	0.70	0.46	Y
6	7.67	1.49	13.67	7.99	3.20	2.08	8.66	3.20	2.30	5.59	72.90	0.68	0.48	Y
7	8.82	1.63	8.53	5.10	2.33	1.61	5.84	2.33	2.07	1.22	13.79	0.36	NSS7	N
8	16.38	1.33	17.65	9.33	3.74	2.33	9.45	3.74	2.38	15.44	94.24	0.60	0.59	Y
9	34.88	1.52	13.71	8.72	3.22	1.92	9.34	3.22	2.15	21.23	60.86	0.50	0.34	Y
10	8.43	0.72	23.43	12.78	2.43	1.81	12.78	2.43	1.81	8.43	100.00	0.70	0.70	Y
11*	4.04	1.58	10.18	5.15	2.06	1.50	7.18	2.06	1.96	0.29	7.27	0.31	0.42	N
12	3.89	1.53	11.95	6.86	2.71	1.67	8.74	2.71	2.04	0.98	25.11	0.62	0.32	N
13	4.29	1.34	17.73	9.64	2.91	2.08	9.90	2.91	2.14	3.88	90.40	0.52	0.48	Y
14	5.27	1.20	12.47	8.24	2.44	1.82	8.41	2.44	1.86	4.87	92.24	0.72	0.71	Y
15	6.04	0.92	12.43	7.09	2.47	1.63	7.11	2.47	1.63	6.00	99.22	0.72	0.71	Y
16	8.90	1.28	13.15	8.07	2.49	1.93	8.09	2.49	1.94	8.86	99.58	0.59	0.59	Y
17	11.56	2.04	12.61	6.95	2.91	2.30	9.80	2.91	2.58	2.17	18.75	0.81	0.52	Y
18	7.58	1.09	16.38	8.10	2.00	1.60	8.35	2.00	1.63	6.66	87.90	0.18	0.05	Y
19	4.36	1.88	10.07	5.33	2.29	1.57	6.36	2.29	2.27	0.01	0.28	0.31	NSS	N
20	10.74	1.23	11.50	6.37	3.97	1.93	6.44	3.97	2.01	9.57	89.07	0.11	0.05	Y

MTV = Metabolic Tumor Volume

HV = Hypoxic Volume

HF = Hypoxic Fraction

R = Pearson Correlation Coefficient

NBD = No Blood Data

NFU = No FDG Uptake

NSS = Not statistically significant

* = Statistical outlier

(T/B > 1.2) = Only analyzed for voxels that have SUV's greater than 1.2 × SUV of Blood

**Table 3:** Results summary for the LNs

Lymph Nodes														
Patient #	FDG MTV	Blood SUV	FDG SUV^max^	FDG SUV^avg^	FMISO SUV^max^	FMISO SUV^avg^	FDG SUV^avg^ (T/B > 1.2)	FMISO SUV^max^ (T/B > 1.2)	FMISO SUV^avg^ (T/B > 1.2)	HV	HF	R	R (T/B > 1.2)	FDG SUVmax within FMISO HV
1	5.05	NBD	13.59	8.16	2.06	1.57	–	–	–	NBD	–	0.46	–	–
2	NFMU	–	–	–	–	–	–	–	–	–	–	–	–	–
3	3.22	0.97	18.75	10.12	1.95	1.45	12.31	1.95	1.70	0.97	30.00	0.48	0.44	Y
4	14.99	4.99	9.37	3.86	2.89	1.56	4.44	2.89	1.96	4.99	33.27	0.45	0.39	Y
5	18.96	0.56	28.55	18.39	2.69	1.95	19.20	2.69	2.59	0.56	2.95	0.42	NSS	N
6	4.82	2.02	8.55	5.11	2.52	1.70	5.60	2.52	2.10	2.02	41.79	0.40	0.15	N
7	37.40	35.67	13.57	8.37	4.55	2.96	8.48	4.55	3.02	35.67	95.36	0.57	0.53	Y
8a	15.36	7.47	10.17	4.80	3.63	1.76	5.47	3.63	2.15	7.47	48.59	0.54	0.37	Y
8b	1.65	1.60	13.45	7.04	3.62	2.55	7.15	3.62	2.58	1.60	97.28	0.88	0.87	Y
9	0.96	0.08	5.62	3.93	1.94	1.59	4.32	1.94	1.88	0.08	8.12	0.48	0.34	N
10	22.35	22.35	20.21	13.93	3.20	2.16	13.93	3.20	2.16	22.35	100.00	0.54	0.54	Y
11*	29.60	9.13	13.74	8.78	2.65	1.75	9.98	2.65	2.09	9.13	30.84	0.51	0.21	Y
12a	25.03	7.82	18.85	13.21	2.60	1.67	14.15	2.60	2.03	7.82	31.22	0.42	0.21	Y
12b	6.87	1.38	18.66	11.79	2.20	1.63	13.83	2.20	1.95	1.38	20.15	0.42	0.11	Y
13	26.94	23.35	14.35	7.58	2.91	1.97	7.79	2.91	2.04	23.35	86.69	0.39	0.23	N
14	11.38	11.12	10.24	7.49	3.34	2.28	7.55	3.34	2.30	11.12	97.74	0.75	0.30	Y
15	25.63	25.59	26.83	17.83	1.85	1.44	17.85	1.85	1.44	25.59	99.81	0.46	0.45	Y
16	23.17	21.91	12.80	8.99	2.43	1.95	9.13	2.43	1.99	21.91	94.56	0.81	0.78	Y
17	3.29	0.51	10.73	5.34	2.85	2.13	7.73	2.85	2.59	0.51	15.58	0.70	0.35	Y
18	NFMU	–	–	–	–	–	–	–	–	–	–	–	–	–
19	21.96	–	12.24	7.25	2.14	1.61	–	–	–	NHV	–	0.31	–	–
20	13.39	13.21	15.35	8.92	3.42	2.27	8.95	3.42	2.28	13.21	98.72	0.40	0.38	Y

MTV = Metabolic Tumor Volume

HF = Hypoxic Fraction

R = Pearson Correlation Coefficient

NFMU = No FMSIO Uptake

NVH = No Hypoxic Volume

NSS = Not statistically significant

(T/B > 1.2) = Only analyzed for voxels that have SUV's greater than 1.2 × SUV of Blood

* = Statistical outlier

**Fig. 2:**
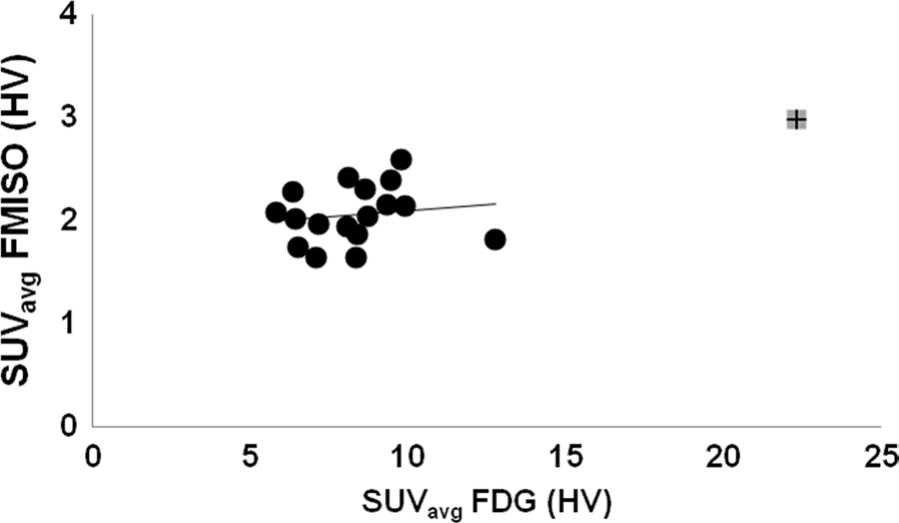
FMISO vs FDG SUVavg of the primary tumors for the HV (defined by a tumor-to-blood SUV ratio greater than 1.2. A moderate Pearson correlation, R = 0.64 (paired t test, P < 0.01), is shown between the two. The greyed cross data point is an outlier that was excluded from the analysis

Comparable results were observed for the HV, with R = 0.61 and R = 0.38 for the average (Fig. 2) and maximum (data not shown) SUVs, respectively.

**Fig. 3:**
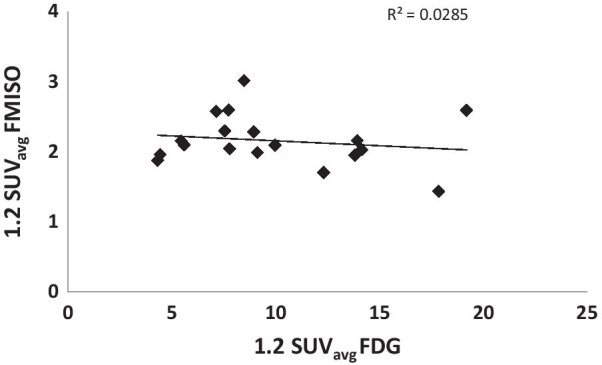
FMISO vs FDG SUVavg of the Lymph Nodes for the HV (defined by a tumor-to-blood SUV ratio greater than 1.2. A weak Pearson correlation, R = 0.17 (paired t test, P < 0.01), is shown between the two

On the other hand, no such correlations between the two radiotracers’ SUVmax’s or SUVavg’s could be established in the case of LNs for either the MTVs (R = 0.08) (data not shown) or the HVs (Fig. 3).

**Fig. 4:**
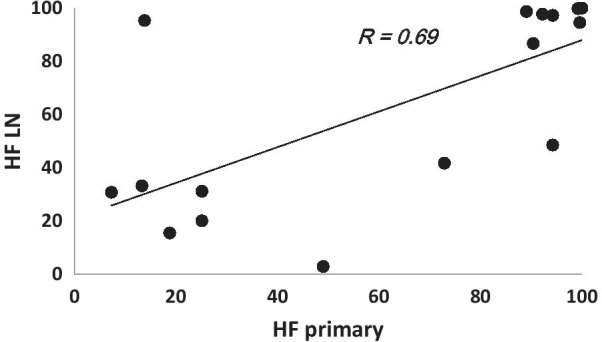
Plot of the correlation between the primary tumors and LNs HF’s. A strong correlation, yet not significant (R = 0.69; paired t test, P = 0.96) between the primaries and LNs oxygenation status seems to exist, which is in agreement with the measurement reported by Becker and in HNSCC using biopsy-based methods [11]

Comparison of the FMISO uptake between primary tumors and LNs showed a moderate correlation (R = 0.56) between the SUV_avg_s of the two for HVs. Weak correlations have been otherwise observed between the corresponding SUVavg’s when using the MTV (R = 0.19), as well as for SUVmax’s whether considering the MTVs (R = 0.18) or only the HVs (R = 0.14). Our results also revealed a moderate correlation (R = 0.67) between the hypoxic fractions of the primary tumors and the LNs (Fig. 4).

Spatially, the position of the voxel with maximum FDG SUV falls within the HVs in 75% and 76% of the lesions in the primary tumors and LNs, respectively. Spatially, in order to comprise the whole hypoxic TV (defined by T/B greater than 1.2), the average FDG hottest volume fraction for all patients was 92% and 97% for the primary tumors and LNs, respectively.

**Fig. 5:**
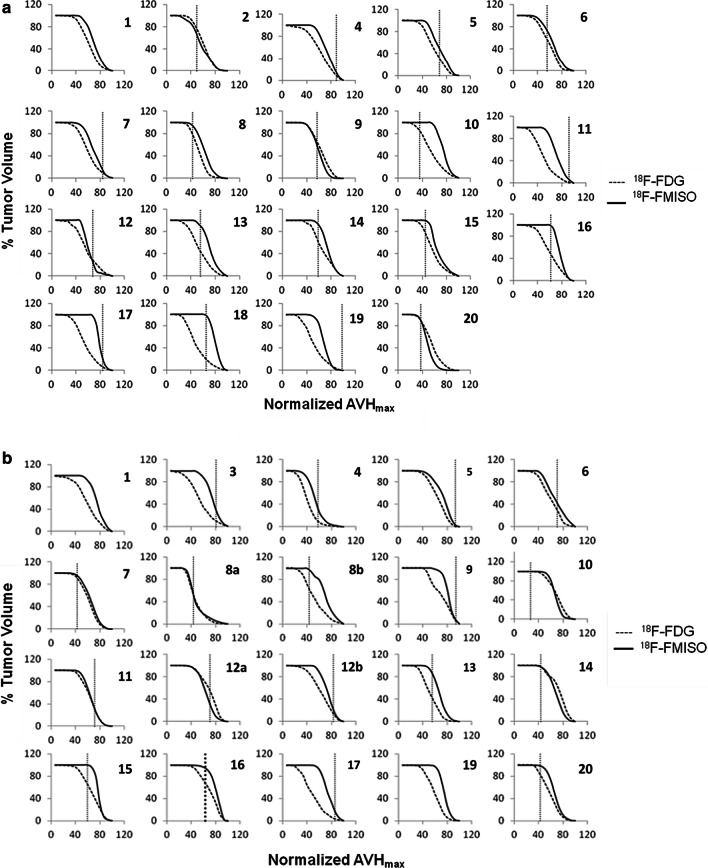

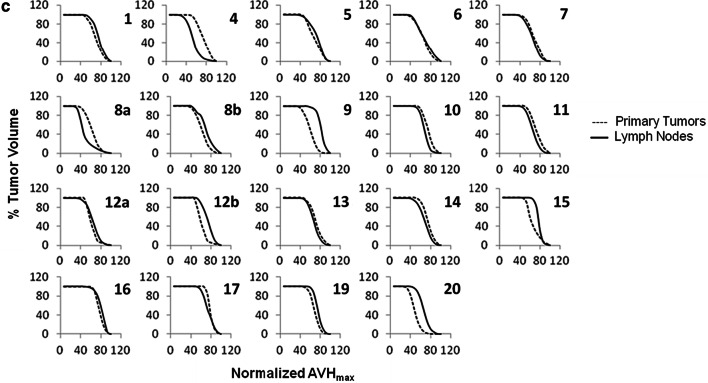
Overlays of the FMISO and FDG activity volume histograms (AVH) of the primary tumors (**a**) and LNs (**b**). Differences between the FDG and FMISO intra-tumoral distribution in the primaries (**a**) and LNs (**b**) by means of a measure of the areas under the corresponding AVH’s appears to be statistically significant. The primary tumors and LNs FMISO AVH’s are shown in figure (**c**). FMISO intra-tumoral distribution in the primaries and LNs appears, however, to be comparable

Finally, comparison of the FDG and FMISO AVHs for the primary tumors and LNs is summarized in Fig. 5a, b, respectively. The areas under the corresponding AVHs, which are used as metrics to measure the homogeneity of the radiotracers intra-tumoral distributions were compared. The differences between the FDG and FMISO areas under the corresponding AVHs were statistically significant (paired t test, P = 0.0001 for the primary and P < 0.0001 for the LNs). Nevertheless, the primary tumors and LNs FMISO AVHs appeared to be comparable (paired t test, P = 0.58) (Fig. 5c).

## Discussion

FMISO PET is a noninvasive imaging technique that has been used to image tumor hypoxia in HNC for more than 15 years [2, 12–15]. However, it remains a research radiotracer requiring custom production and an approved IND (Investigative New Drug) and IRB. Glucose metabolism is regulated by many pathways including hypoxia. The use of FDG PET to infer information of hypoxia is an attractive one, which has been investigated in multiple studies with the ultimate goal of obviating the need for hypoxia-specific imaging probes [5, 16].

Several pre-clinical studies suggested a correlation between glucose metabolism, as measured with FDG, and hypoxia. In cell lines studies, Minn and co-workers showed a mean increase of [^3^H]-FDG uptake of 120% and 46% under anoxic conditions over that measured at a baseline 20% atmospheric oxygen concentration for two head and neck squamous cell carcinoma cell lines, UT-SCC-5 and UT-SCC-20A, respectively [17]. Pre-clinically, using a Dunning prostate tumor model, Pugachev and co-workers showed a positive correlation between FDG uptake and hypoxia defined by pimonidazole staining [18]. Wyss and colleagues also found similar results [19]. However, in the clinical setting, data are limited and discordant, and most studies showing a moderate to weak correlation between the two radiotracers. For example, Zimny et al. showed FDG uptake to be independent of the tumor oxygenation status which was not correlated with the corresponding pO_2_-polarography measurements [16]. The same group, however, showed a strong correlation between FMISO tumor-to-muscle ratio and the frequency of pO_2_ readings less than or equal to 5 mmHg [16]. Likewise, in a study that included 12 HNC patients who underwent FDG and FMISO exams prior to radiotherapy, Thorwarth et al. showed an ambiguous correlation between the two radiotracers [14]. More promising results were reported by Rajendran and co-workers, where a mean correlation of 0.62 between FDG and FMISO concentrations was observed in a study that included 26 HNC patients, based on analyzing primary tumor sites [13].

Due to the wide availability of FDG, it would be attractive to assess whether a correlation between the two radiotracers, i.e. FMISO and FDG, exists, and hence whether the FDG may be used as a surrogate for tumor hypoxia. In this study, we have investigated the correlation between tumor hypoxia and glucose metabolism, as imaged by FMISO and FDG PET, respectively, using a voxel-wise analysis as well as global semi-quantitative parameters (SUVmax and SUVmean). We also reported on the general similarity of the two radiotracers’ distributions within the MTV by means of comparing the corresponding activity volume histograms (AVHs). Nineteen primary tumors and 20 metastatic LNs from a total of 20 HNSCC patients were included in the analysis. Primary tumors and LNs that did not show FDG or FMISO uptake were excluded. The primary tumor sites and the metastatic LNs were analyzed independently in case the correlation between FDG and FMISO differed, as was previously reported by Komar et al. [20]. Quantitatively, the correlation between FDG and FMISO uptakes was also investigated using two criteria; first, this was done using the MTV, and then second, using only the HV which was defined by a tumor-to-blood ratio (T/B) threshold greater than 1.2, as was initially proposed by Rajendran and colleagues [5]. As clarified above, this threshold value of 1.2 was an operational definition and was based on the observation that < 5% of voxels in whole-body FMISO PET images corresponding to normal tissues exceeded this value.

Voxel-by-voxel FDG-FMISO analysis showed a strong correlation R (> 0.7) in ~ 68% of the primary tumors and in 40% of the LNs. However, these correlation coefficients can be significantly impacted by good correspondence between the less relevant low-activity regions, whereas the potential correlation between the high activity FDG and FMISO regions is more important. Restricting the analysis to the HVs defined by a T/B > 1.2 resulted in a decrease in the correlation coefficient values so that only 39% of the primary tumors and 22% of the LNs resulted in R values > 0.7. Inaccuracies in patient setup and consequent image registration based on mutual information of the CT component of the FMISO1 and FMISO2 PET/CT studies also affect the strength of the correlation [21]. The error in patient setup was previously estimated by Hong *et al* [22]*.* in head and neck cancer patients to be in the order of 6.97 mm (*i.e.*, approximately two PET pixels).

Although FDG uptake is related to expression and activity of specific GLUT and glycolytic activity in the tumor, it is also affected by several other factors such as radioresistance, proliferation [23], cell density [24], and hypoxia [25, 26]. There have been multiple attempts to validate the feasibility of FDG-based dose-painting to improve local tumor control by radiation therapy. In cases where the HVs are contained within the high FDG-uptake regions within the TV, the consequence of such a dose-painting strategy would be dose escalation to the HV in addition to other hyperglycolytic regions within the tumor. Our results indicate that the FDG threshold required in order to include the entire HV would necessitate inclusion of more than 90% of the hypermetabolic TV. This encompasses most of the FDG avid volume, and suggests that the hypoxic voxels are dispersed throughout the FDG-avid TV. In another word, our data suggest that it is not possible to inclusively escalate the dose to the HVs within the tumor by just boosting the dose to the hottest sub-regions within the tumor characterized with high FDG SUV. Even though this strategy might be successful for selected patients, it was not found to be universally applicable.

In a previous study in which a cohort of HNSCC, Nehmeh et al. showed that the tumor hypoxia can be spatially dynamic within the target volume, thus suggesting the presence of acute hypoxia [21]. In order to successfully escalate the radiotherapy dose to the hypoxic volume confirmation of its spatial stability within the tumor volume, i.e. chronic rather than acute hypoxia, is a pre-requisite. In another study, Lee et al. investigated the prognostic values of pre- and mid- treatment FMISO PET in a cohort HNSCC undergoing platinum-based chemo-radiation [27]. The authors showed excellent loco-regional control despite evidence of detectable hypoxia in the pre-treatment FMISO PET studies. They also showed that treatment outcome was independent of the hypoxic status of tumors at mid-treatment [27]. In contrast, Nicolay et al. showed a correlation between tumor hypoxia dynamics, as measured by FMISO PET, and, treatment response and outcome in HNSCC patients undergoing chemoradiation [28]. In another study, Widenmann et al. showed the potential of multi-parametric MRI as a surrogate to FMISO PET to longitudinally monitor tumor hypoxia in HNSCC patients undergoing chemoradiation [29]. This can have significant impact on patients management especially to the large availability of MRI, as well as due to its higher spatial resolution compared to PET.

Here, we also investigated the potential utility of FDG for predicting tumor hypoxia. Quantitative comparison between FDG and FMISO uptakes showed, on average, a moderate correlation (P < 0.01) between the two markers’ SUV_avg_ for the primary tumors for both the MTVs, and the HVs (Fig. 2). This is in agreement with the results of Rajendran et al. in a study of 26 HNC primary tumors [13]. Discrepant results, however, were reported by Thorwarth et al., who did not find any correlation between FDG and FMISO uptake in 12 primary HNC tumors [14]. This lack of correlation may be due to the limited range of FDG SUVmax values (mean = 9.53; range 7.84 to 12.07) [14], which may require greater statistical power (i.e. many more lesions) in order to deduce a possible correlation between them. In contrast, the study by Rajendran et al. included patients with a wide range of FDG SUVmax (mean = 10.9; range 2.9 to 25.4) [2]. The latter is comparable to the range in our study (mean = 14.7; range 7.6 to 32.2). In contrast to our findings in primary tumors, we did not detect any correlation between FDG and FMISO SUV in lymph node metastases (Fig. 3). This was also noted by Gagel et al. [4]. The FMISO uptake appeared to be independent of the corresponding FDG uptake (regression slopes ~ 0) in LNs (Fig. 3), perhaps indicating that biologic processes contributing to glucose uptake (e.g., perfusion, hypoxia, proliferation) contribute to variable degree to the FDG signal in primary tumors versus nodal metastases. Similarity in the characteristics of the primary tumors and LNs microenvironments are vital if both are to be managed the same way, otherwise a more complicated lesion-based treatment approach would be necessary. Comparing the FMISO uptake between primary tumors and LNs on a patient-by-patient basis resulted in weak correlations between the corresponding SUV_avg_s (R = 0.18) when including the MTVs as well as for the SUV_max_’s (R = 0.19 and R = 0.14 for the MTVs and HVs, respectively). Similar results have been reported for other noninvasive hypoxia markers including EF5 and fluoroazomycin arabinoside (FAZA) [20]. However, this is in contradiction with a report that showed a correlation between the oxygenation status of the primary tumor and that of the metastatic LNs using biopsy-based methods. A moderate correlation (R ~ 0.56) was, however, observed between the primary tumors and LNs SUV_avg_s for the HVs. Moreover, a moderate correlation yet statistically insignificant (R = 0.69; P = 0.96) has been shown between the HFs of the primary tumors and LNs (Fig. 4). Becker and co-workers reported similar results on the correlation between the oxygenation status of the primary tumor and that of the metastatic LNs in HNSCC using biopsy-based methods [11].

Both FDG and FMISO SUV’s are known to change as a function of time post-injection. The differences in uptake times post-injection, in both FDG (range 50 min to 180 min) and FMISO (patients cohort-1 range 114 min to 195 min; patients cohort-2 range 135 min to 181 min), may therefore be considered as a major source of uncertainty in this study. To our knowledge, correlation of intratumoral distributions of FDG (FMISO) between different time points post-injection is yet to be investigated. Till then, it would difficult to predict the effect of the ranges of times post-injection considered in this study on the correlations between the two radiotracers. Finally, qualitative comparison of FDG and FMISO intra-tumor distributions by means of the AVH’s showed the latter to be more homogeneously distributed in both primary and LNs (supplemental Fig. 5a, b). Using the area under the AVH as a measure of homogeneity, the differences between those of FDG and FMISO were shown to be statistically significant (paired t test, P = 0.0001 for the primary and P < 0.0001 for the LNs). Nevertheless, the primary tumors and LNs FMISO AVHs appeared to be comparable (paired t test, P = 0.58) (supplemental Fig. 5c). Finally, 7 out of the 20 subjects included in this study were HPV-positive. However, clinical data suggested there is no significant difference in the level, nor distribution of hypoxia in HPV-positive and HPV-negative tumors, as measured by a 15-gene hypoxia classifier [30] and FMISO PET [31].

One major limitation of this study is the small number of subjects included, which was a result of the complexity of the protocol. This could be the reason for weak to moderate correlations that were observed. Analysis of a larger cohort of subjects will be necessary before the findings in this study can be confirmed. Another limitation is the inaccuracy of image registration which can impact the accuracy of the correlation between the FDG and FMISO correlation, mainly in the voxel-wise analysis.

### Conclusion

A moderate correlation was observed between FDG and FMISO distributions in the primary HNSCC tumors, but not for the LNs. There was a moderate correlation observed between the individual HFs of the primary tumors and their metastatic LNs. Our findings do not show a universal correlation between FDG and FMISO to exist for all tumors, and therefore FDG PET images cannot be used by themselves as a universal surrogate to identify or predict intra-tumor hypoxia. However, combining FDG PET data with contrast enhanced CT data, to provide supplementary spatial information on tissue perfusion, has been suggested as a path to improve the derivation of hypoxia information based on clinical standard of care scans [32].

## Data Availability

Anonymized data can be made available upon request.

## References

[1] Le QT (2006). An evaluation of tumor oxygenation and gene expression in patients with early stage non-small cell lung cancers. Clin Cancer Res.

[2] Rajendran JG (2006). Tumor hypoxia imaging with [F-18] fluoromisonidazole positron emission tomography in head and neck cancer. Clin Cancer Res.

[3] Piert M (1999). Introducing fluorine-18 fluoromisonidazole positron emission tomography for the localisation and quantification of pig liver hypoxia. Eur J Nucl Med.

[4] Gagel B (2004). pO(2) polarography versus positron emission tomography [F-18] fluoromisonidazole, [F-18]-2-fluoro-2 '-deoxyglucose) - An appraisal of radiotherapeutically relevant hypoxia. Strahlenther Onkol.

[5] Rajendran JG (2003). [F-18]FMISO and [F-18]FDG PET imaging in soft tissue sarcomas: correlation of hypoxia, metabolism and VEGF expression. Eur J Nucl Med Mol Imaging.

[6] Nicolay NH (2020). Correlative analyses between tissue-based hypoxia biomarkers and hypoxia PET imaging in head and neck cancer patients during radiochemotherapy-results from a prospective trial. Eur J Nucl Med Mol Imaging.

[7] Airley RE, Mobasheri A (2007). Hypoxic regulation of glucose transport, anaerobic metabolism and angiogenesis in cancer: novel pathways and targets for anticancer therapeutics. Chemotherapy.

[8] Kim JW, Gao P, Dang CV (2007). Effects of hypoxia on tumor metabolism. Cancer Metastasis Rev.

[9] Zhao S (2005). Biologic correlates of intratumoral heterogeneity in 18F-FDG distribution with regional expression of glucose transporters and hexokinase-II in experimental tumor. J Nucl Med.

[10] ImageJ., *Available from: *http://rsb.info.nih.gov/ij*. Accessed June 2006.*

[11] Becker A (1998). Oxygenation of squamous cell carcinoma of the head and neck: Comparison of primary tumors, neck node metastases, and normal tissue. Int J Radiat Oncol Biol Phys.

[12] Eschmann SM (2005). Prognostic impact of hypoxia imaging with 18F-misonidazole PET in non-small cell lung cancer and head and neck cancer before radiotherapy. J Nucl Med.

[13] Rajendran JG (2004). Hypoxia and glucose metabolism in malignant tumors: Evaluation by [F-18]fluoromisonidazole and [F-18]fluorodeoxyglucose positron emission tomography imaging. Clin Cancer Res.

[14] Thorwarth D (2006). Combined uptake of [18F]FDG and [18F]FMISO correlates with radiation therapy outcome in head-and-neck cancer patients. Radiother Oncol.

[15] Hendrickson K (2011). Hypoxia imaging with [F-18] FMISO-PET in head and neck cancer: potential for guiding intensity modulated radiation therapy in overcoming hypoxia-induced treatment resistance. Radiother Oncol.

[16] Zimny M (2006). FDG–a marker of tumour hypoxia? A comparison with [18F]fluoromisonidazole and pO2-polarography in metastatic head and neck cancer. Eur J Nucl Med Mol Imaging.

[17] Minn H, Clavo AC, Wahl RL (1996). Influence of hypoxia on tracer accumulation in squamous-cell carcinoma: in vitro evaluation for PET imaging. Nucl Med Biol.

[18] Pugachev A (2005). Dependence of FDG uptake on tumor microenvironment. Int J Radiat Oncol Biol Phys.

[19] Wyss MT (2006). NanoPET imaging of [(18)F]fluoromisonidazole uptake in experimental mouse tumours. Eur J Nucl Med Mol Imaging.

[20] Komar G (2008). 18F-EF5: a new PET tracer for imaging hypoxia in head and neck cancer. J Nucl Med.

[21] Nehmeh SA (2008). Reproducibility of intratumor distribution of (18)F-fluoromisonidazole in head and neck cancer. Int J Radiat Oncol Biol Phys.

[22] Hong TS (2005). The impact of daily setup variations on head-and-neck intensity-modulated radiation therapy. Int J Radiat Oncol Biol Phys.

[23] Vesselle H (2000). Lung cancer proliferation correlates with [F-18]fluorodeoxyglucose uptake by positron emission tomography. Clin Cancer Res.

[24] Dooms C (2009). Association Between 18F-Fluoro-2-Deoxy-D-Glucose Uptake Values and Tumor Vitality: Prognostic Value of Positron Emission Tomography in Early-Stage Non-small Cell Lung Cancer. J Thorac Oncol.

[25] van Baardwijk A (2007). The maximum uptake of (18)F-deoxyglucose on positron emission tomography scan correlates with survival, hypoxia inducible factor-1alpha and GLUT-1 in non-small cell lung cancer. Eur J Cancer.

[26] Sattler UGA, Mueller-Klieser W (2009). The anti-oxidant capacity of tumour glycolysis. Int J Radiat Biol.

[27] Lee N (2009). Prospective trial incorporating pre-/mid-treatment [18F]-misonidazole positron emission tomography for head-and-neck cancer patients undergoing concurrent chemoradiotherapy. Int J Radiat Oncol Biol Phys.

[28] Nicolay NH (2019). Correlative Analyses between Tissue-Based Hypoxia Biomarkers and Hypoxia PET Imaging in Head and Neck Cancer Patients during Radiochemotherapy: Results from a Prospective Trial. Int J Radiat Oncol Biol Phys.

[29] Wiedenmann, N., et al., *The utility of multiparametric MRI to characterize hypoxic tumor subvolumes in comparison to FMISO PET/CT. Consequences for diagnosis and chemoradiation treatment planning in head and neck cancer.* Radiother Oncol, 2020. **150**: p. 128–135. 10.1016/j.radonc.2020.06.013 32544609

[30] Toustrup K (2012). Gene expression classifier predicts for hypoxic modification of radiotherapy with nimorazole in squamous cell carcinomas of the head and neck. Radiother Oncol.

[31] Trinkaus ME (2014). Correlation of p16 status, hypoxic imaging using [18F]-misonidazole positron emission tomography and outcome in patients with loco-regionally advanced head and neck cancer. J Med Imaging Radiat Oncol.

[32] Crispin-Ortuzar M (2018). Predicting hypoxia status using a combination of contrast-enhanced computed tomography and [F-18]-Fluorodeoxyglucose positron emission tomography radiomics features. Radiother Oncol.

